# Anti-Proliferative Effect and Induction of Apoptosis in Androgen-Independent Human Prostate Cancer Cells by 1,5-Bis(2-hydroxyphenyl)-1,4-pentadiene-3-one

**DOI:** 10.3390/molecules20023406

**Published:** 2015-02-17

**Authors:** Kamini Citalingam, Faridah Abas, Nordin H. Lajis, Iekhsan Othman, Rakesh Naidu

**Affiliations:** 1 Jeffery Cheah School of Medicine & Health Sciences, Monash University Malaysia, Jalan Lagoon Selatan, 47500 Bandar Sunway, Selangor, Malaysia; E-Mails: kaminicitalingam@gmail.com (K.C.); iekhsan.othman@monash.edu (I.O.); 2 Laboratory of Natural Products, Faculty of Science, Universiti Putra Malaysia, 43400 UPM Serdang, Selangor, Malaysia; E-Mail: faridah@food.upm.edu.my; 3 Department of Food Science, Faculty of Food Science and Technology, Universiti Putra Malaysia, 43400 UPM Serdang, Selangor, Malaysia; E-Mail: nordinlajis@gmail.com

**Keywords:** androgen-independent, prostate cancer, diarylpentanoids, cytotoxicity, apoptosis

## Abstract

Curcumin has poor *in vivo* absorption and bioavailability, highlighting a need for new curcumin analogues with better characteristics in these aspects. The aim of this study is to determine the anti-cancer properties of four selected curcumin analogues, on the cytotoxicity, proliferative and apoptotic effects on androgen-independent human prostate cancer cells (PC-3 and DU 145). Initial cytotoxicity screening showed MS17 has the highest cell inhibitory effect, with EC_50_ values of 4.4 ± 0.3 and 4.1 ± 0.8 µM, followed by MS13 (7.5 ± 0.1 and 7.4 ± 2.6 µM), MS49 (14.5 ± 1.2 and 12.3 ± 2.3 µM) and MS40E (28.0 ± 7.8 and 30.3 ± 1.9 µM) for PC-3 and DU 145 cells, respectively. Time-dependent analysis also revealed that MS13 and MS17 displayed a greater anti-proliferative effect than the other compounds. MS17 was chosen based on the high selectivity index value for further analysis on the morphological and biochemical hallmarks of apoptosis. Fluorescence microscopy analysis revealed apoptotic changes in both treated prostate cancer cells. Relative caspase-3 activity increased significantly at 48 h in PC-3 and 12 h in DU 145 cells. Highest enrichment of free nucleosomes was noted at 48 h after treatment with MS17. In conclusion, MS17 demonstrated anti-proliferative effect and induces apoptosis in a time and dose-dependent manner suggesting its potential for development as an anti-cancer agent for androgen-independent prostate cancer.

## 1. Introduction

Prostate cancer is one of the most commonly diagnosed cancers and a leading cause of cancer death in men. Prostate cancer is a clinically heterogeneous disease which harbors multiple genetic abnormalities accumulated during the progression of the disease. The cellular mechanisms contributing to prostate cancer involve a multistep process that includes the inactivation of tumor suppressor genes and the dysregulation of several oncogenic pathways. Although the exact causes of prostate cancer remain unclear, it has been well documented that androgens (testosterone and 5α-dihydrotestosterone, DHT) play an important role in the physiological development of the normal prostate and prostate cancer [1]. Exposure to higher levels of androgens or overexpression/mutation of androgen receptor often leads to rapid proliferation of prostate cancer cells and, almost all the patients eventually relapse with tumors that become androgen-independent [2,3]. At this stage, the cancer cells begin to metastasize to various organs, ultimately causing the death of the patient.

At the initial stage prostate tumors respond to hormonal therapies, and currently available chemotherapeutic drugs are successful in treating these localized, androgen-dependent cancer. These tumors eventually progress to androgen-independent forms that are refractory to these therapies and treatment thus remains hindered and represents a challenge for the clinical oncologist [4]. This has led to little overall improvement of morbidity and mortality, and therefore novel drugs are required to treat hormone-resistant prostate cancer. Alterations of several molecular pathways are required for the development of androgen independence and the dilemma is how to develop the most effective therapeutic drugs that are required to treat hormone-resistant prostate cancer. Tumor cells activate multiple pathways to survive under castration levels of androgens [5,6,7].

Curcumin (diferuloylmethane), an active yellow pigment, is a major active component of turmeric. It is isolated from the rhizomes of *Curcuma longa* and has been widely used for decades in the Asian countries, particularly in South Asia. The chemistry of curcumin induces biological effects that allow it to influence multiple cell signaling pathways, giving it anti-inflammatory, antioxidant, chemo-preventive, chemotherapeutic, anti-mutagenic, anti-metastatic and anti-angiogenic properties. Several studies have demonstrated that curcumin has a number of anticancer properties [8,9] and it was found to be highly cytotoxic towards several tumor cell lines. At the molecular level there is evidence that curcumin inhibits the growth of a variety of human cancer cell lines *in vitro* by cell cycle arrest and induction of apoptosis through inhibition of several protein and/or pathways such as cyclin, cyclin-dependent kinase, NF-κB, protein kinase C and mitogen-activated protein kinase (MAPK). It also suppresses pro-inflammatory signaling by inhibiting the expression and activity of cyclooxygenase-2 (COX-2) [10]. Curcumin has been reported to have anti-prostate cancer activity *in vitro* and *in vivo* in both androgen-dependent and androgen-independent prostate cancer [11,12]. It has been shown to inhibit many targets such as transcription factors, receptors, intracellular kinases, cytokines, and growth factors in prostate epithelial cells associated with cancer formation and progression [13]. Its ability to treat hormone-refractory prostate cancer suggests that curcumin could be a potential candidate as androgen-independent agent against prostate cancer.

Curcumin was demonstrated to have a wide spectrum of pharmacological properties with an absence of systemic toxicity. However, it has poor bioavailability, which has been determined in both animal models and humans [14], limits its clinical application as a potential anticancer agent. This limitation has led researchers to develop a variety of synthetic analogues of curcumin with similar safety profiles and increased activity, but improved bioavailability. Several analogues of curcumin with different bioactivities through modification of the molecular structure have resulted in the development of potential anti-cancer candidates that target various cancers, including prostate cancer [15,16,17,18,19,20,21,22,23,24,25,26,27,28]. Therefore curcumin analogues can be potentially used to treat hormone-refractory prostate cancer which has the worst prognosis with lower survival rates.

**Figure 1:**
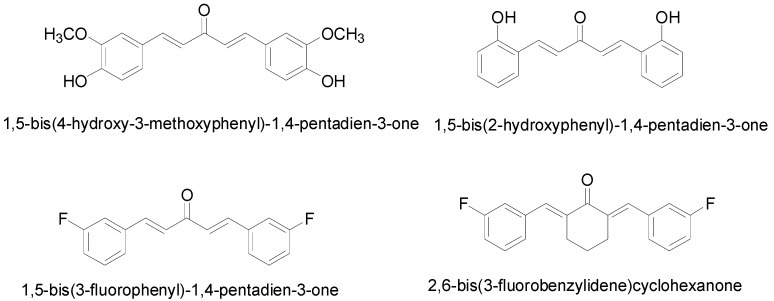
Molecular structures of diarylpentanoid derivatives.

Initial screening of 29 curcumin-like diarylpentanoids with two identical aromatic ring regions separated by five carbon spacers on colorectal and cervical cancer cells have revealed that four compounds, namely 1,5-bis(4-hydroxy-3-methoxyphenyl)-1,4-pentadiene-3-one (MS13), 1,5-bis(2-hydroxyphenyl)-1,4-pentadiene-3-one (MS17), 1,5-bis(3-fluorophenyl)-1,4-pentadiene-3-one (MS40E) and 2,6-bis(3-fluorobenzylidene)cyclohexanone (MS49) (Figure 1) were effective at inhibiting cancer cell viability and growth.

A separate study done by Nagaraju and colleagues reported two potential curcumin analogues, EF31 and UBS109 which displayed a significantly higher growth inhibitory effect in pancreatic cancer cells *in vitro* and *in vivo* compared to curcumin [29]. On the other hand, a similar study on MS17, labeled ca27 by Fajardo *et al*., reported anti-androgenic activities in androgen-dependent prostate cancer cells but the effect in androgen-independent prostate cancer cells was not evaluated [30]. Therefore in this study, we evaluated these four compounds for their cytotoxicity, anti-proliferative effect and ability to induce apoptosis in androgen-independent prostate cancer cells, mainly PC-3 and DU 145 cells. These cells were chosen as a model of androgen-independent human prostate cancer cells due to the absence of androgen receptor (AR) mRNA and protein expression [31,32] at the transcriptional level as a result of epigenetic modification of AR [33,34,35]. This may play a crucial role in the development of hormone independence in a subset of prostate cancers that do not express AR. Hence, the focus of this research is to identify the anti-cancer properties of the selected diarylpentanoids on the androgen-independent metastatic prostate cancer cells as potential chemotherapeutic agents for the treatment of prostate cancer.

## 2. Results and Discussion

### 2.1. Cytotoxicity Analysis of the Diarylpentanoids on the PC-3 and DU 145 Cell Lines

**Figure 2:**
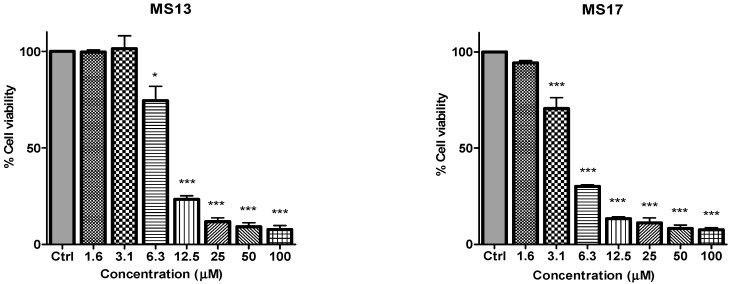

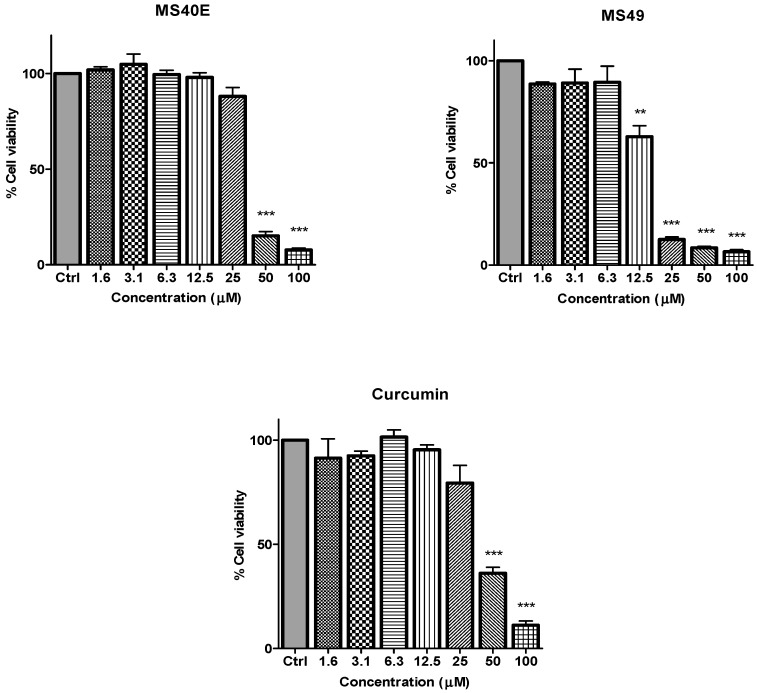
The inhibitory effects of cell viability by curcumin, MS17, MS13, MS40E and MS49 in PC-3 cell line compared to untreated sample (Ctrl). Results are expressed as means ± S.E. from three independent experiments. Percentage cell viability and comparison between data sets performed using ANOVA. * *p* < 0.05, ** *p* < 0.01 and *** *p* < 0.001 indicates statistically significant differences between the means of values obtained with treated *vs.* untreated cells.

**Figure 3:**
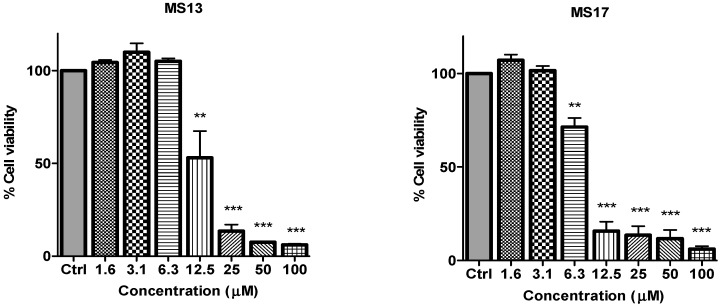

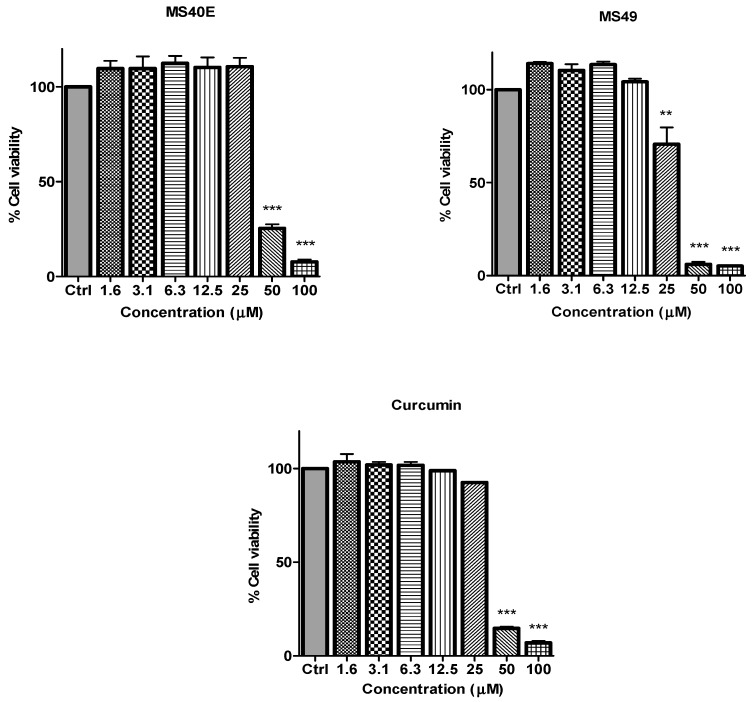
The inhibitory effects of cell viability by curcumin, MS17, MS13, MS40E and MS49 in DU 145 cell line compared to untreated sample (Ctrl). Results are expressed as means ± S.E. from three independent experiments. Percentage cell viability and comparison between data sets performed using ANOVA. ** *p* < 0.01 and *** *p* < 0.001 indicates statistically significant differences between the means of values obtained with treated *vs.* untreated cells.

To determine the *in vitro* dose-dependent cytotoxicity effect of the curcumin analogues MS13, MS17, MS40E, and MS49, we performed a prostate cancer cell viability assay. The ability of the diarylpentanoids to inhibit growth of the two widely studied and well characterized androgen independent metastatic human prostate cancer cell lines PC-3 and DU 145 were assessed using the MTT assay. This colorimetric assay is based on the ability of mitochondrial enzymes of live cells to reduce MTT to formazan salt. Cells treated with curcumin and DMSO only was used as positive and negative controls, respectively. Among the tested compounds, MS17 showed the strongest dose and time-dependent cytotoxicity effect in both cell lines with a significant decrease in cell viability at 3.1 µM onwards in PC-3 and 6.3 µM onwards in DU 145 cells. This is followed by MS13 with a significant decrease in cell viability at doses of 6.3 µM and 12.5 µM in PC-3 and DU 145 cell respectively. However, the other compounds MS49 and MS40E exhibited moderate growth inhibitory effect in both cell lines. MS49 revealed a significant decrease in cell viability at doses of 12.5 µM and 25 µM onwards in PC-3 and DU 145 cells respectively whereas, MS40E at a dose of 50 µM onwards in both cell lines. Curcumin, the parent compound displayed a significant cell inhibitory effect at a dose of 50 µM (Figure 2 and Figure 3). Based on the cytotoxicity study of all the compounds on both cell lines, the EC_50_ value of each compound was measured. As shown in Table 1, MS17 showed a significantly lower EC_50_ value in both the cancer cell lines. Treatment with MS17 stated an EC_50_ value of 4.4 ± 0.3 µM in PC-3 and 4.1 ± 0.8 µM in DU 145 compared to that of curcumin, the parent compound. In both cell lines, the order of potencies was MS17 > MS13 > MS49 > MS40E > Curcumin (Table 1).

**Table 1:** EC_50_ values of curcumin and its analogues on prostate cancer cell lines (PC-3 and DU 145) and normal cell lines (WI-38 and WRL 68).

Compounds	EC_50_ Values (µM) of Compounds on Cell Lines			
	PC-3	DU 145	WI-38	WRL-68
MS13	7.5 ± 0.1	7.4 ± 2.6	9.4 ± 2.3	8.7 ± 0.2
MS17	4.4 ± 0.3	4.1 ± 0.8	5.2 ± 1.2	5.5 ± 0.5
MS40E	28.0 ± 7.8	30.3 ± 1.9	28.4 ± 1.7	30.3 ± 2.0
MS49	14.5 ± 1.2	12.3 ± 2.3	19.4 ± 7.0	13.1 ± 0.4
* Curcumin	35.9 ± 2.9	32.5 ± 1.4	27.9 ± 2.9	28.7 ± 3.2

* Curcumin was used as the positive control. Results are shown as mean ± standard deviation (SD) from three independent experiments.

The cytotoxicity effects of all compounds were further evaluated for their toxicity against normal human liver epithelia (WRL-68) and human lung fibroblast (WI-38) cell lines. Interestingly, all four compounds showed lower cytotoxicity against the normal cell lines. As a whole, these results revealed that all four compounds showed an improved dose-dependent growth inhibitory effect on PC-3 and DU 145 cells compared to curcumin. These results also suggest that MS17 has the strongest cell inhibitory effect on PC-3 and DU 145 cells. Furthermore, in order to evaluate the most toxic compound towards cancer cells, the corresponding selectivity index (*SX*) values were determined. Based on the calculated *SX* values, MS17 showed comparably high *SX* value in both the cancer cell lines (Table 2) compared to curcumin which exhibited the lowest *SX* value in both the cancer cell lines.

**Table 2:** Selective index (*SX*) values of compounds on each tested prostate cancer cell lines and normal cell lines.

Compounds	WI-38 (Normal Human Lung Fibroblast Cell)		WRL68 (Normal Human Epithelial Hepatocytes)	
	PC-3	DU 145	PC-3	DU 145
MS13	124.8	127.2	115.1	117.3
MS17	117.4	126.3	124.9	134.3
MS40E	101.4	93.5	108.3	100.0
MS49	134.3	158.6	90.4	106.8
Curcumin	77.5	85.8	79.8	88.2

*SX* values > 100 indicates that the cytotoxic effect of the tested compound is greater towards cancer cells.

### 2.2. Determination of the Anti-Proliferative Effect of the Compounds on the Prostate Cancer Cells

To further confirm the potential of the compounds inhibiting cell growth in both cancer cell lines, a BrdU cell proliferation assay was performed. The cell proliferation percentage was measured based on the incorporation of the bromodeoxyuridine (BrdU) into the DNA of the proliferating cells. The percentage of reduction or decrease of cell proliferation is proportional to the percentage reduction of BrdU incorporation. The anti-proliferative study which is a time-dependent assay evaluates the approximate duration taken for growth inhibition to be initiated. Elucidation of anti-proliferative effect of the compounds was based on the fact that relatively low drug concentration induced considerable growth inhibition of the prostate cancer cell lines. Overall, treatment with the compounds resulted in a concentration and time-dependent reduction in cell proliferation rate in PC-3 and DU 145 cell lines. At all three time points, MS17 and MS13 showed a greater anti-proliferative effect compared to the other compounds.

**Figure 4:**
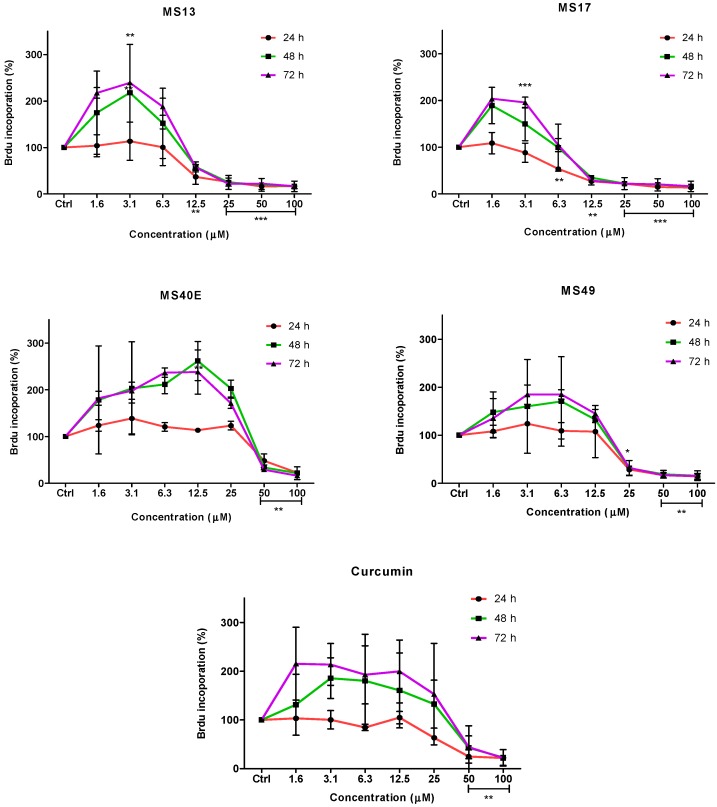
Anti-proliferative effect of MS13, MS17, MS40E, MS49 and curcumin on PC-3 cells at 24, 48 and 72 h. Results are expressed as means ± S.E. from three independent experiments. Percentage BrdU incorporation and comparison between data sets performed using ANOVA. * *p* < 0.05, ** *p* < 0.01 and *** *p* < 0.001 indicates statistically significant differences between the means of values obtained with treated *vs.* untreated cells (control).

In PC-3 cells, treatment with MS13 caused a significant reduction in cell proliferation at 12.5 µM onwards for 24, 48 and 72 h. The reduction at 24 h was approximately 60%–80% and 40%–80% at 48 and 72 h compared to the control. However, MS17 showed a significant anti-proliferative activity at 6.3 µM onwards for 24 h treatment with approximately 50%–90% reduction in cell proliferation compared to the control. At 48 and 72 h, the reduction in cell proliferation was significant at 12.5 µM onwards with approximately 80%–90% reduction. As for treatment with MS49, results showed a significant anti-proliferative activity at 25 µM onwards for all three time points. However, MS40E and curcumin showed a moderate anti-proliferative activity at 50 µM onwards for 24, 48 and 72 h compared to the control (Figure 4).

**Figure 5:**
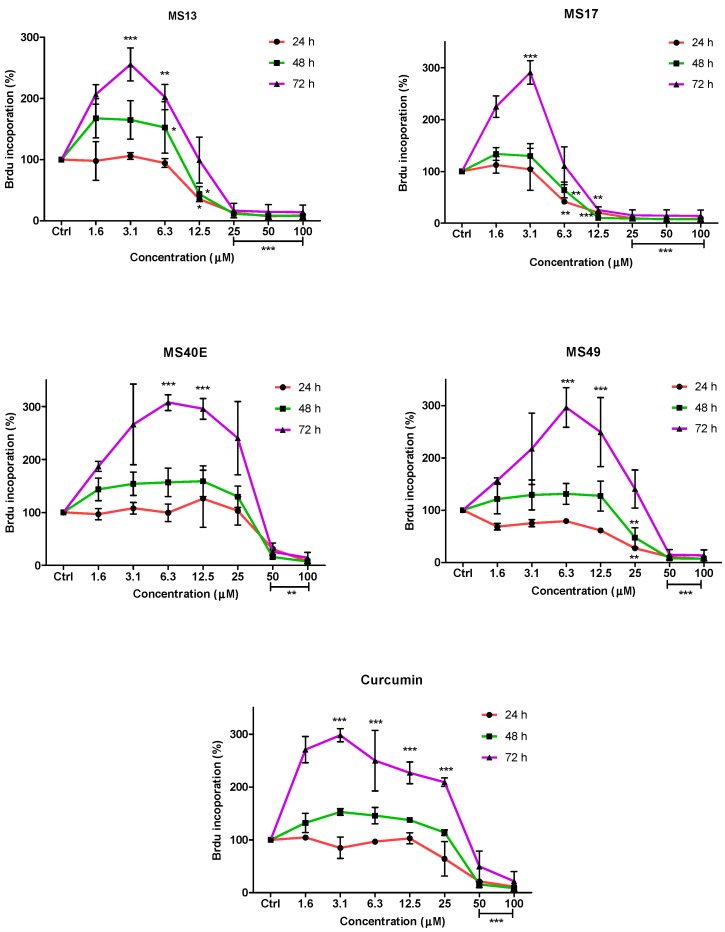
Anti-proliferative effect of MS13, MS17, MS40E, MS49 and curcumin on DU 145 cells at 24, 48 and 72 h. Results are expressed as means ± S.E. from three independent experiments. Percentage BrdU incorporation and comparison between data sets performed using ANOVA. * *p* < 0.05, ** *p* < 0.01 and *** *p* < 0.001 indicates statistically significant differences between the means of values obtained with treated *vs.* untreated cells (control).

On the other hand, treatment with MS13 in DU 145 cells showed a significant anti-proliferative activity at 12.5 µM onwards for 24 and 48 h, with approximately 60%–90% reduction in cell proliferation. However, at 72 h the reduction was noted to be significant at 25 µM onwards compared to the control cells. In MS17 treatment, a significant anti-proliferative activity was seen at 6.3 µM onwards for both the time points compared to the control cells with approximately 60%–90% reduction at 24 h and 30%–90% reduction at 48 h. For 72 h, treatment with MS17 caused a significant decrease in cell proliferation at 12.5 µM onwards. Nonetheless, MS49 showed a significant anti-proliferative activity at 25 µM onwards for both 24 and 48 h treatment, but at 72 h the reduction was significant at 50 µM onwards. However, MS40E and curcumin showed a similar anti-proliferative activity which was significant at 50 µM for all three time points (Figure 5). These results indicate that treatment with MS13 and MS17 had a greater anti-proliferative activity in both the cell lines compared to curcumin as a greater reduction of BrdU incorporation suggests a higher anti-proliferative activity.

### 2.3. Induction of Apoptosis

The mode of killing exerted by most of the effective anti-cancer agents is through the induction of apoptosis. Therefore, the induction of apoptosis was evaluated using several techniques. For a more comprehensive analysis, only MS17 was selected for further analysis on the apoptosis assays based on the selective cytotoxic activity of this compound. The apoptosis effect of MS17 was accessed using three concentrations based on EC_50_, 2X EC_50_ and 3X EC_50_ values at appropriate time points.

#### 2.3.1. Morphological Observation of Cancer Cell Lines by Acridine Orange (AO)—Ethidium Bromide (EB) Double Staining Technique Using Fluorescence Microscope

The morphological assessment of cell death was performed in order to confirm that MS17 treatment of PC-3 and DU 145 prostate cancer cells was inducing apoptosis rather than necrosis. Treated cells that were double stained with acridine orange/ethidium bromide (AO/EB) were examined under a fluorescence microscope.

**Figure 6:**
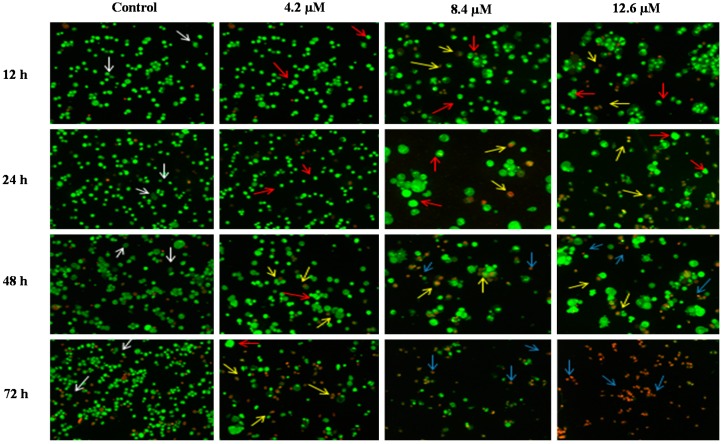
Detection by fluorescent microscopy of acridine orange/ethidium bromide double stained PC-3 cells treated with MS17 for 12, 24, 48 and 72 h. Untreated viable cells are uniformly pale-green (white arrow); Early apoptotic cells shows characteristic loss of membrane integrity and chromatin condensation, stained bright-green to yellow (red arrow); Late apoptotic cells stained yellow-orange or red colour, with a condensed or fragmented chromatin (yellow arrow); Necrotic cells showed bright orange-red in appearance (blue arrow). Magnification 100×.

**Figure 7:**
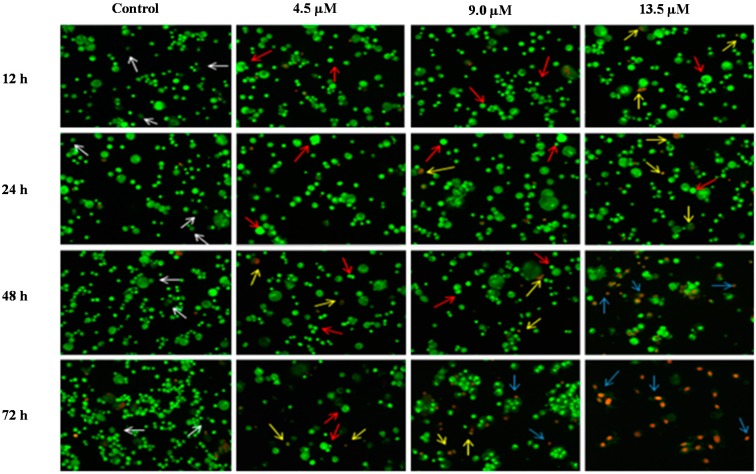
Detection by fluorescent microscope of acridine orange/ethidium bromide double stained DU 145 cells treated with MS17 for 12, 24, 48 and 72 h. Untreated viable cells are uniformly pale-green (white arrow); Early apoptotic cells shows characteristic loss of membrane integrity and chromatin condensation, stained bright-green to yellow (red arrow); Late apoptotic cells stained yellow-orange or red colour, with a condensed or fragmented chromatin (yellow arrow); Necrotic cells showed bright orange-red in appearance (blue arrow). Magnification 100×.

AO is a crucial dye which can stain nucleus DNA in an intact cell membrane, whereas EB stains cells that have lost membrane integrity. Under fluorescent emission, AO is taken up by both the viable and non-viable cells emitting a green fluorescence, whereas EB is taken up only by the non-viable cells emitting red fluorescence if intercalated into the DNA strand. Hence, the live cells have a normal green nucleus whereas the early apoptotic cells have a bright green nucleus with condensed or fragmented chromatin and late apoptotic cells show condensed or fragmented yellow-orange or red chromatin whereas necrotic cells display a bright orange or red uniform nucleus. The morphological characteristics of the MS17 treated cells were photographed and depicted in Figure 6 and Figure 7.

The morphological observation in the cell nuclei of MS17-treated PC-3 and DU 145 cells showed various morphological alterations when compared to the untreated cells (control). The control cells appeared to be intact oval shape and the nucleus were stained uniformly pale-green due to the AO dye. However, MS17-treated cells showed typical signs of apoptosis such as cell shrinkage, formation of apoptotic bodies and membrane blebbing [36]. In both cell lines, as the treatment doses increase, there was a significant decrease in the appearance of viable cells (white arrow).

In PC-3 cells, treatment with 4.5 and 9.0 µM of MS17 at 12 and 24 h displayed higher intensity of bright green fluorescent cells (red arrow) compared to the control, revealing an early apoptotic cells. However, at 48 and 72 h there was a mixed population of cells stained bright green to yellow, indicative of early and late apoptosis (red and yellow arrow). At 72 h, 13.5 µM MS17-treated cells displayed a high proportion of necrotic cells. Necrotic cells which showed loss of membrane integrity emit orange-red fluorescence due to the leakage of EB dye into the nuclei (blue arrow). In DU 145 cells, treatment with 4.2 µM of MS17 at 12 and 24 h showed a higher number of cells stained bright green fluorescent (red arrow) compared to 48 and 72 h which displayed a mixed population of bright green to yellow cells (red and yellow arrow). Treatment with 8.4 and 12.6 µM at 12 and 24 h revealed a mixed population of early and late apoptotic cells, stained bright green to yellow (red and yellow arrow) compared to 48 and 72 h which displayed a higher intensity of necrotic cells, stained orange-red (blue arrow). The morphological characteristic of necrotic cells can be seen clearly in DU 145 cells at 72 h with the treatment of the highest concentration. Collectively, the number of cells stained bright orange-red were generally very low in all MS17-treated cells which suggest that cell death occurred mostly due to apoptosis rather than necrosis.

#### 2.3.2. Quantification of Apoptotic and Necrotic Cells

**Figure 8:**
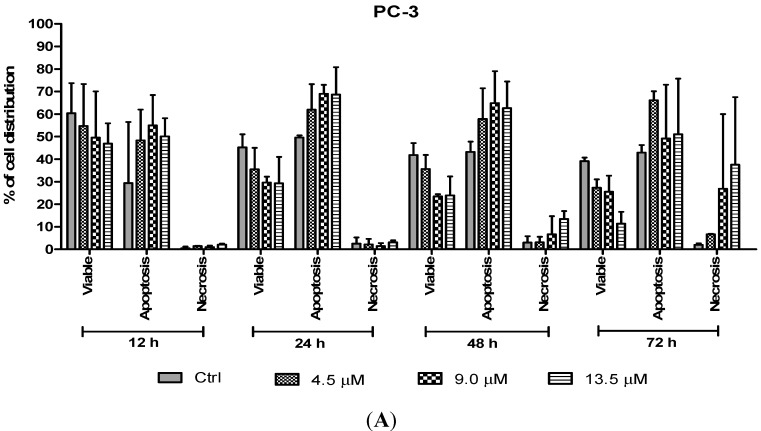

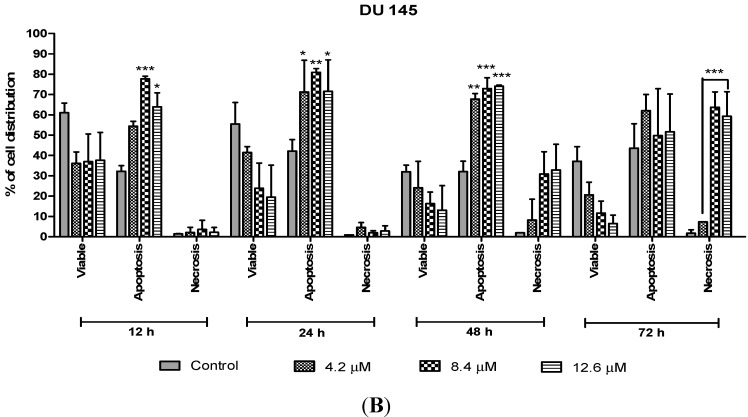
Percentage of cell population in (**A**) PC-3 cells and (**B**) DU 145 cells treated with MS17 for 12, 24, 48 and 72 h. Treated and non-treated cells were double stained with AO/EB and a minimum of 200 cells were counted per sample and the percentage of cells from each population (viable, apoptosis and necrosis) was calculated. Samples were run in triplicates and comparable results were obtained from three independent experiments. Comparison between data sets was performed using ANOVA. * *p* < 0.05, ** *p* < 0.01 and *** *p* < 0.001 indicates statistically significant differences between the means of values obtained with treated *vs.* control.

The percentages of apoptotic and necrotic cells were also determined. For the purpose of analysis, early and late apoptosis were combined to indicate an apoptotic activity. As observed in Figure 8, the percentage of viable cells decreased upon exposure to longer incubation times in both cell lines. Between time points, there was an increase of apoptosis in all concentrations compared to the untreated samples (control). The results showed a comparatively higher percentage of cells undergoing apoptosis at 24 and 48 h as treatment doses increase in both cell lines. PC-3 cells treated with doses of 4.5 µM, 9.0 µM and 13.5 µM at 24 and 48 h induced approximately 60%–70% apoptotic cells. Although apoptosis was relatively high at 48 h, necrotic cells were noted to increase at this time point. Treatment with all three doses at 48 and 72 h showed an increase approximately between 5% and 15% of necrotic cells compared to 12 and 24 h with less than 5% of necrotic cells.

On the other hand, treatment with doses of 4.2, 8.4 and 12.6 µM in DU 145 cells significantly induced approximately 70%–80% apoptotic cells at 24 and 48 h compared to the control cells. However, treatment with a dose of 4.2 µM at 48 h induced approximately 10% necrotic cells to between 30% and 35% necrotic cells at doses of 8.4 µM and 12.6 µM. Strikingly, at 24 h necrotic cells remained low, with approximately lesser than 5% necrotic cells at all examined doses. In summary, MS17 increased apoptosis activity the highest at 24 h after treatment with a low percentage of necrosis. Staining of acridine orange and ethidium bromide in MS17 treated cells provides morphological evidence that MS17 induces apoptosis for both cell lines time and dose-dependently.

#### 2.3.3. Quantification of Relative Caspase-3 Activity

Caspase-3, a member in the family of cysteine proteases is one of the critical enzymes involved in apoptosis. Caspase-3 is synthesized as an inactive pro-enzyme that is responsible for apoptosis execution. Activation of the caspase-3 activity causes cleavage of the key substrate within the cell to execute the apoptotic process which induces DNA fragmentation and morphological changes such as cell blebbing, cell shrinkage, nuclear fragmentation and chromatin condensation. As caspase-3 enzyme are crucial executors in this process, the apoptotic effects of MS17 treated prostate cancer cells were investigated by studying the activity of caspase-3 enzyme.

**Figure 9:**
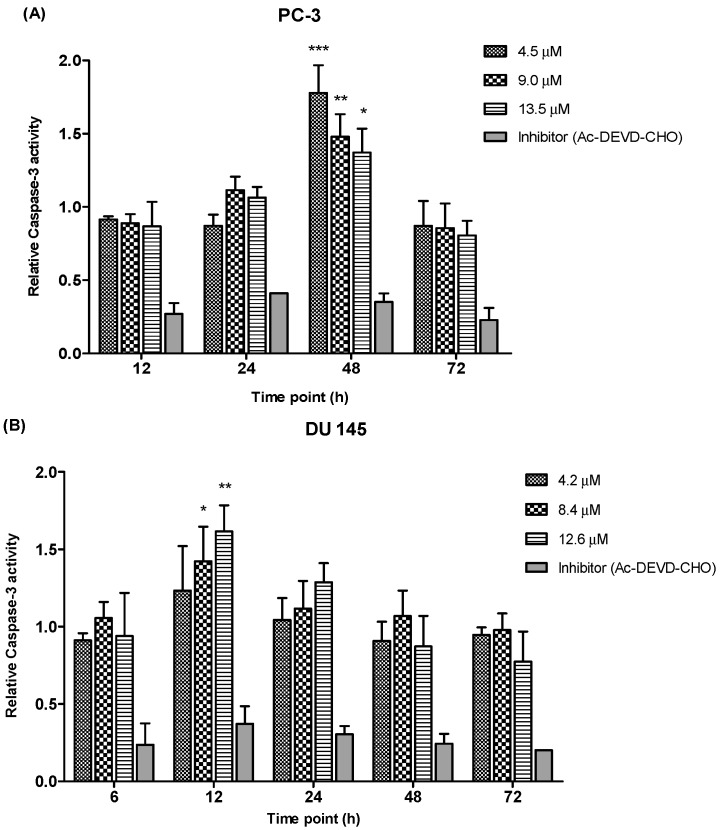
Relative caspase-3 activity in (**A**) PC-3 and (**B**) DU 145 cells treated with MS17 for 12, 24, 48 and 72 h. Results are expressed as mean ± S.E. from three independent experiments and comparison between data sets was performed using ANOVA. * *p* < 0.05, ** *p* < 0.01 and *** *p* < 0.001 indicates statistically significant differences between the means of values obtained with treated *vs.* control. Caspase-3 inhibitor, Ac-DEVD-CHO was used as a control to determine the inhibitory activity of caspase-3.

We determined the relative caspase-3 apoptotic activity in both cell lines at various time points (6, 12, 24, 48 and 72 h as appropriate) and at three different concentrations, the EC_50_, 2 × EC_50_ and 3 × EC_50_ values, which were 4.5 µM, 9.0 µM and 13.5 µM for PC-3 and 4.2 µM, 8.4 µM and 12.6 µM for DU 145 cells. Relative caspase-3 activity was measured based on caspase-3 activity from the treated cells against the untreated cells (control). As shown in Figure 9, PC-3 cells showed a significant increase of caspase-3 activity at 48 h time point following treatment with 4.5, 9.0 and 13.5 µM of MS17. However, there were no significant differences of caspase-3 activity noted between different concentrations at 12, 24 and 72 h. In DU 145 cells an increase in caspase-3 activity was noted at 12 h after treatment with MS17. Caspase-3 activity was significant at 8.4 and 12.6 µM compared to 4.2 µM. However, there were no significant differences of caspase-3 activity between different concentrations at 6, 24, 48 and 72 h. Treatment with the caspase-3 inhibitor, Ac-DEVD-CHO in both cell lines showed relatively low expression levels at all time points for both cell lines, confirming inhibition of caspase-3 activity. 

Overall, these results indicate that MS17 induces apoptosis by caspase-3 activity in both cell lines with PC-3 demonstrated a dose-dependent decrease at 48 h while DU 145 displayed a dose-dependent increase at 12 h.

#### 2.3.4. Detection of Free Nucleosomes (ELISA)

To further elucidate the type of cell death, another important biochemical hallmark of apoptosis is the detection of DNA fragments/fragments of genomic DNA (mono- and oligonucleosomes) in the cytoplasm of apoptotic cells. In this study, the induction of apoptosis by the treatment of MS17 was measured through the combination of anti-histone and anti-DNA capture and detection antibodies through the ELISA method. The prostate cancer cell lines were treated with three different concentrations of MS17 and incubated over 24, 48 and 72 h and the presence of free nucleosomes (mono- and oligonucleosomes) in the cytoplasm were measured based on the enrichment factor.

**Figure 10:**
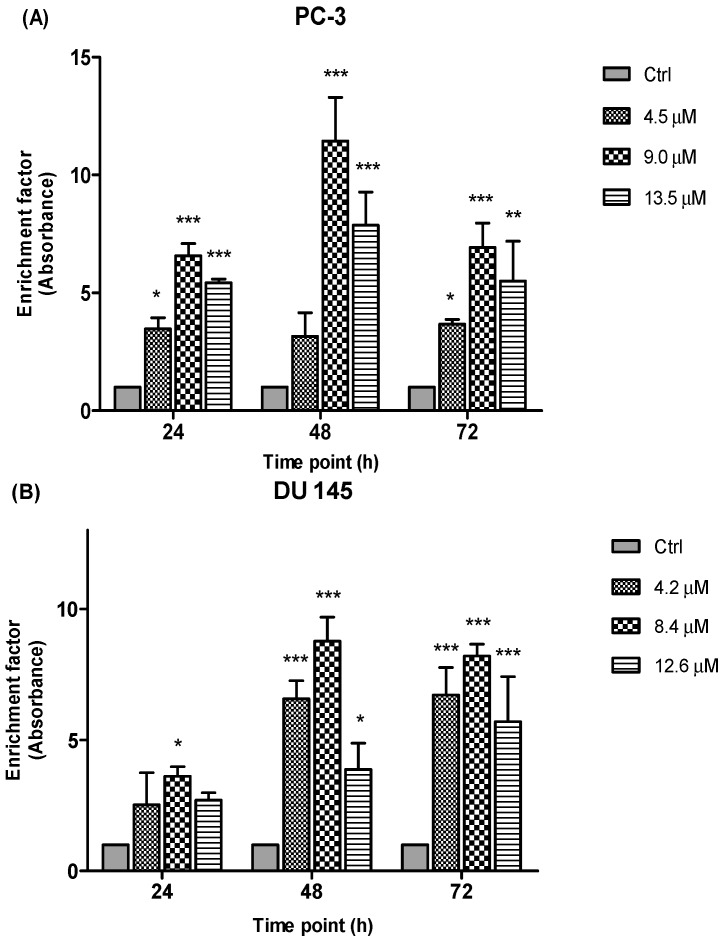
Enrichment of nucleosomes in MS17-treated (**A**) PC-3 and (**B**) DU 145 cells at 24, 48 and 72 h incubation. Results are expressed as mean ratio ± S.E. of the absorbance from cells treated with MS17 relative to absorbance of untreated cells and comparison between data sets was performed using ANOVA. * *p* < 0.05, ** *p* < 0.01 and *** *p* < 0.001 indicates statistically significant differences between the means of values obtained with treated *vs.* control. Samples were run in duplicates and comparable results obtained from two independent experiments.

Results (Figure 10) are shown as ratio of absorbance calculated according to the formula introduced in the previous section. The enrichment factor value is an indicative of apoptotic activity for all the concentrations and time points. Following the exposure of PC-3 and DU 145 cells to three different concentrations of MS17 treatment mainly 4.5 µM, 9.0 µM and 13.5 µM for PC-3 and 4.2 µM, 8.4 µM and 12.6 µM for DU 145 cells at 24, 48 and 72 h respectively, apoptotic activity was noted at all examined time points and concentrations of MS17 compared to the corresponding untreated cells (control). As seen in Figure 10, the results of the PC-3 cells revealed a significant increase in nucleosomal fragments which was measured as an enrichment factor at doses of 9.0 and 13.5 µM for all three time points. However, greater increase in nucleosomes was noted at 48 h with the treatment dose of 9.0 µM. In DU 145 cells, treatment with MS17 at dose of 8.4 µM revealed a significant increase in nucleosomal fragments compared with 4.2 and 12.6 µM at all time points. Interestingly, a greater increase in nucleosomal fragmentation was noted at 48 h compared to the control cells. Hence, these results indicate that MS17 induced apoptotic cell death in PC-3 and DU 145 cells.

### 2.4. Discussion

Curcumin, the multi-target natural product, has extensively been used as a lead compound to design and synthesize analogues for the potential treatment of prostate cancer. This compound has been proven to inhibit many targets in prostate epithelia cells with an importance in cancer formation and progression. However, it has previously been reported that curcumin exhibits poor bioavailability *in vitro* and *in vivo* [14]. In light of these efforts, our laboratory has been studying the effectiveness of using selected synthetic diarylpentanoid curcumin analogues as agents for prostate cancer therapy. In this study, we reported the cytotoxic activity of these compounds and the mode of apoptosis induced by a selected compound on the prostate cancer cell lines, mainly PC-3 and DU 145 cells. To the best of our knowledge, this study represents a first report of the cytotoxic and apoptosis activity of these analogues on human androgen-independent prostate cancer cells.

Based on the initial cytotoxicity screening of the diarylpentanoids on the two prostate cancer cells, compound MS17 showed an improved cytotoxicity and growth inhibitory effect in a dose-dependent manner at lower concentrations compared to the parent compound, curcumin. The low EC_50_ value (Table 1) supported the results, indicating that MS17 had the most significant toxicity effect in both cell lines, followed by MS13, MS49, MS40E and lastly curcumin. This result was also supported by the selectivity index value which showed that MS17 is more selective towards cancer cells (Table 2). However, screening of the compounds on the normal cell lines with comparable doses provided evidence that the normal cell lines are more resistant to the growth suppressive effects of this compound compared to prostate cancer cells, suggesting that it is relatively less toxic to normal cells. A study done by Fajardo and team [30] reported that the compound MS17, which was labeled as ca27, is able to inhibit the growth of prostate cancer cells at a concentration below that typically used for curcumin. Furthermore, this compound was also found to exert anti-androgenic effect in androgen-dependent prostate cancer cells. However, its effect on androgen-independent prostate cancer cells was not evaluated. It is believed that certain modification to the analogue structure from its parent compound has resulted in a better cytotoxicity activity. Introduction of suitable alkoxy groups and modification of the length of the linker between the biphenyl rings were shown to have greater growth suppressive potential, compared to curcumin [37].

Dose and time-dependent cytotoxicity of the compounds on the prostate cancer cell lines were also observed. An anti-proliferative study revealed a time-dependent growth inhibitory effect of all compounds. The anti-proliferative study was measured based on the incorporation of BrdU into the DNA of the proliferating cancer cells during the S phase of the cell cycle [38]. As shown in the results (Figure 4 and Figure 5), an increase in the exposure time to the compounds appeared to have a significant anti-proliferative effect in both the cell lines. Furthermore, treatment with MS17 demonstrated greater anti-proliferative effect at all three time points compared to the other compounds and curcumin in both cell lines. This observation could be due to the characteristic of the cell lines. Although both cell lines are metastatic form of prostate cancer cells, DU 145 cells which expresses a mutated p53 protein is relatively more resistant to treatment compared to PC-3 cells which harbors a wild-type p53 protein. However, both dose and time-dependent assays showed selectivity for greater growth inhibitory effect in both cells lines were more significant through the treatment of MS17.

Induction of apoptosis, a programmed cell death, has been identified as the primary strategy in cancer chemotherapy and a gold standard for an anti-cancer candidate [39]. In order to investigate whether the cytotoxic effect and inhibition of cell growth were related with the apoptotic event, the effect of the compounds on apoptosis induction were studied via various morphological and biochemical methods. However, for a more comprehensive analysis, only MS17 was selected from the other compounds for further analysis on the apoptosis assays using three concentrations, EC_50_, 2 × EC_50_ and 3 × EC_50_ values. Apoptosis results revealed that MS17 could be mediating cell death through apoptosis.

Apoptotic cells were first recognized through the characteristic changes in their morphology, particularly changes in their nuclei. Fluorescence microscopy analysis on MS17-treated cells double stained with acridine orange (AO) and ethidium bromide (EB) provided morphological evidence that MS17 induced apoptosis in both cell lines. The fluorescence microscopy analysis revealed the early stages of apoptosis which are characterized by the shrinkage of cells, blistering and membrane blebbing [40,41]. As shown in the time and dose-dependent treatment, both cell lines triggered nearly 50% apoptosis after 24 h in all doses of MS17 treatment. DU 145 cells stained bright green to yellow between 12 and 24 h compared to PC-3 cells between 24 and 48 h. The increase in the percentage of cells stained bright orange-red at 72 h treatment of both cell lines, identified by nucleus staining with EB may correspond partly to the appearance of necrotic cells where the plasma membrane becomes permeable. Overall, MS17 has the capability to induce apoptosis, evident by the morphological changes in DU 145 and PC-3 cells, dose and time-dependently.

The ability of MS17 to induce apoptosis is also supported by measuring the activation of caspase-3 and detection of nucleosomal fragments in both the cell lines. A primary biochemical event marking the start of apoptosis is the activation of caspases. Caspase-3 is one of the critical enzymes involved in apoptosis. It is involved in the activation cascade of caspases responsible for apoptosis execution. Caspase-3 functions as an executioner caspase that cleaves various substrates including PARP. It is observed that, activation of caspase-3 in both cell lines at specific doses and time point correlated with the early morphological signs of apoptosis. In DU 145 cells relative caspase-3 activity resulted in a dose-dependent increase at 12 h after treatment and reduced thereafter. However, in PC-3 cells caspase-3 activity increased significantly at 48 h after treatment and declined gradually at 72 h, thus validating the results from the fluorescence microscopic quantification analysis. Collectively, it is suggested that the cell death episode in both the cancer cells could be partly mediated through the activation of caspase-3, the promoter of apoptosis.

Besides, detection of nucleosomal fragments as a result of DNA degradation has also been widely used as a hallmark of apoptosis processes [42]. Nuclear fragmentation was measured based on the enrichment of nucleosomes (mono- or oligonucleosomes) in cytoplasm of apoptotic cells. In the present study, enrichment of nucleosomes was detected highest at 48 h after MS17-treatment in both cell lines, which correlates with the morphological signs of apoptosis process. There is a body of evidence that suggest nucleosomal fragments in apoptotic cells are associated with the activation of caspase-3 activity. In the event when caspase-3 is activated, caspase-3 cleaves the cytoskeletal proteins and stimulates the caspase-activated DNase which eventually enters the nucleus and degrades DNA leading to the death of the cell [43]. Hence, taken together, these results verify a preferential apoptotic process than necrotic effect of MS17 on PC-3 and DU 145 cells.

Overall, MS17 revealed a significant dose-dependent and time-dependent effect in inducing growth inhibition in PC-3 and DU 145 cells. Based on the apoptosis data it is evident that MS17 mediates cell death through apoptosis in both the prostate cancer cell lines. Apoptosis induction is a highly desirable characteristic of treatment strategy for cancer control as it opens a new strategy in the search of anti-cancer drugs. Hence, it is of an importance that in a tested compound, apoptosis is induced rather than necrosis as a cell death module.

## 3. Experimental Section

### 3.1. Source and Maintenance of Cell Lines

Two androgen-independent metastatic human prostate cancer cell lines, PC-3 and DU 145 cells and two normal cell lines, WI-38 (human lung fibroblast cells) and WRL 68 (human epithelial hepatocytes) were purchased from American Type Culture Collection (ATCC, Rockville, MD, USA) and maintained using appropriate media. PC-3 and DU 145 cells were grown in Ham’s F-12 Medium (F-12K) and Eagle’s minimum essential medium (EMEM), respectively, whereas both the normal cell lines were grown in EMEM media (ATCC). All cell lines were supplemented with 10% foetal bovine serum (FBS, Gibco, Grand Island, NY, USA) and 1% penicillin (100 U/mL)/streptomycin (100 µg/mL) (Gibco). Cells were grown on a monolayer culture in a tissue culture flask (Nunc, Roskilde, Denmark) and observed routinely under inverted microscope for contaminations. The medium was changed every 2–3 days and the cells were passaged weekly using accutase (Gibco) as the cell detachment solution. Cells were left to grow till it reached a confluency of 80%–90% at 37 °C in a humidified incubator with 5% CO_2_.

### 3.2. Preparation of Curcumin Analogues

All curcumin analogues: 1,5-bis(4-hydroxy-3-methoxyphenyl)-1,4-pentadiene-3-one (MS13), 1,5-bis(2-hydroxyphenyl)-1,4-pentadiene-3-one (MS17), 1,5-bis(3-fluorophenyl)-1,4-pentadiene-3-one (MS40E) and 2,6-bis(3fluorobenzylidene)cyclohexanone (MS49) were synthesized by coupling the appropriate aromatic aldehyde with acetone and cyclohexanone under base catalyzed aldol condensation conditions, using a 1:2 ratio of ketone to aldehyde [44]. All compounds were characterized based on the analysis of spectroscopic data and comparison of these data with those of related compounds. The molecular structures of the diarylpentanoids are shown in Figure 1.

### 3.3. Cytotoxicity Assay

Cell viability was determined by the MTT [3-(4,5-dimethylthiazol-2-yl)-2,5-diphenyltetrazolium bromide] assay, as previously described by Mosmann [45] with modifications. Briefly, cells were detached from the tissue culture flask and pelleted by centrifugation at 1000 rpm for 5 min. Then, the density of the viable cells was counted using a haemocytometer. Cells were then plated in a 96-well flat bottom micro-titer plate (Nunc) at a concentration of 10^4^ cells/mL and incubated in a 5% CO_2_ incubator at 37 °C overnight to allow the cells to adhere to the bottom of the wells. After 24 h, the media was aspirated off and replaced with fresh media containing sample compounds (curcumin and curcumin analogues) of seven different concentrations ranging from 1.56 µM–100 µM and incubated at 37 °C, 5% CO_2_ for 72 h. Separately, the sample compounds were prepared in a stock solution of 50 mM in 100% dimethyl sulfoxide (DMSO, Molecular Biology Grade, Sigma-Aldrich, St. Louis, MO, USA) and diluted in the culture media to a working concentration of 200 µM. Curcumin, the parent compound which was used as the positive control was also prepared in the similar manner. A two-fold serial dilution was done for each sample in triplicates down the columns of the plate, yielding a final volume and concentration of 100 µL in 0.2% DMSO in each wells. Following treatment, 50 µL MTT solutions (0.5 mg/mL in PBS) was added to each well and incubated for another 4 h at 37 °C in a humidified 5% CO_2_ incubator. The supernatant was replaced with 100 µL of DMSO and the optical density (OD) was measured at 570/650 nm using a micro-plate ELISA reader (Bio-Rad Laboratories, Hercules, CA, USA). The percentage of viable cells was calculated based on the ratio of the corrected absorbance of sample-treated cells to the corrected absorbance of the control (0.2% DMSO-treated cells) multiplied by 100. The percentage of cell viability was determined as follows:

$$\%\text{ Cell viability}=\;\frac{\text{OD of treated cells }}{\text{OD of control}}\;\times 100$$

For each compound tested, the EC_50_ value was generated from the log dose-response curves for each cell line using the Graphpad Prism version 5.04 for Windows (Graphpad Software, La Jolla, CA, USA). EC_50_ value represents the concentration at which the tested compound caused 50% growth inhibition, averaged from the three experiments. Cytotoxicity of each tested compound is expressed as the EC_50_ value. Based on the EC_50_ values, selectivity index (*SX*) value was also determined. Selectivity index is a special parameter applied as a selectivity indicator of tested compounds towards cancer cells [46]. The *SX* represents the ratio of EC_50_ value obtained in toxicity testing using the normal cell lines to the EC_50_ values of the toxicity testing of the cancer cells, and then multiplied by 100. All the compounds were assessed for their potential toxicity against normal, non-cancerous cells. The *SX* value above 100 indicates that the cytotoxicity effect of the tested compound is greater towards cancer cells whereas, *SX* value of 100 or below would suggest that the tested concentration of the compound for achieving therapeutic effect is similar to or lower than the concentration causing toxic effect to normal cells. The *SX* values were calculated using the following equation:

$$\mathrm{SX}\;=\;\frac{{\text{EC}}_{50}\;(\text{normal cells})}{{\text{EC}}_{50}\;(\text{cancer cells})}\;\times \;100$$

### 3.4. Bromodeoxyuridine (BrdU) Cell Proliferation Assay

The anti-proliferative effects of compound on treated cells, was measured using the BrdU cell proliferation assay kit (Roche Applied Science Diagnostics, Indianapolis, IN, USA) according to the manufacturer’s protocol. The assay was based on incorporation of BrdU during DNA synthesis in proliferating cells. Cells were plated at a concentration of 10^4^ cells/mL in a 96-well microtiter plate and incubated overnight. The cells were treated with compounds and curcumin (concentrations ranging from 1.56 µM–100 µM) as positive control for 24, 48 and 72 h respectively. Then, BrdU labeling solution (5 µL, diluted by 1/100 in culture media) was added to each well and incubated for 2 h at 37 °C. The media was replaced with fix/denaturing solution (100 µL) and incubated for 30 min at room temperature. Then, the fix/denaturing solution was removed by tapping off and followed by addition of 50 µL anti-BrdU antibody (50 µL, diluted by 1/100 in dilution buffer) to each well and further incubated for another 90 min at room temperature. Next, the cells were washed 3 times with wash buffer and substrate solution (50 µL) was added to each well. The plate was left for 10 min at room temperature in the dark and the absorbance was measured at a wavelength of 370 nm with a reference wavelength of 492 nm using a micro-plate ELISA reader (Bio-Rad). The percentage of BrdU incorporation was determined based on the ratio of absorbance of sample treated-cells to absorbance of control (untreated cells/DMSO only) multiplied by 100.

### 3.5. Morphological Analysis of Apoptotic Cells by Acridine Orange (AO)—Ethidium Bromide (EB) Double Staining

Cell morphological changes were assessed by the differential staining DNA-intercalate fluorescent dyes acridine orange (AO) and ethidium bromide (EB). Cells (3 × 10^5^) were seeded in a 6-well tissue culture plates, treated with MS17 at various concentrations (EC_50_, 2 × EC_50_, 3 × EC_50_) and incubated for 12, 24, 48 and 72 h time intervals. After incubation, cell pellets were suspended in 50 µL of cold PBS. To each samples, 10 µL of cell suspension was added with 1 µL of AO/EB dye mixture (1 part of 100 µg/mL of AO in PBS; 1 part of 100 µg/mL of EB in PBS) prior to microscopic examination. Ten microliter of the cell suspension mixed with the dye mixture was placed on a glass microscopic slide covered with a cover slip and was observed and photographed with a fluorescence microscope using a fluorescein filter (BX41, Olympus, Center Valley, PA, USA). A minimum of 200 total targeted cells were counted per sample and the percentage of cells from each population (viable, early apoptosis, late apoptosis and necrosis) was calculated. The experiment was conducted in triplicates. Untreated cells containing DMSO alone were used as negative control.

### 3.6. Caspase-3 Activity Assay

Caspase-3 activity was determined using the caspase-3 colorimetric assay kit (Sigma-Aldrich) according to manufacturer’s protocol. The assay is based on spectrophotometric detection of *p*-nitroanilide (pNA) a colored molecule, after its cleavage from the labeled substrate DEVD-pNA by the activity of caspase-3 enzyme. PC-3 and DU 145 cells were treated with MS17 at different concentrations (EC_50_, 2 × EC_50_, 3 × EC_50_) at various time points, 6, 12, 24, 48 and 72 h as appropriate. Following treatment, cells were washed with PBS and suspended with lysis buffer (50 mM HEPES, pH 7.4, 5 mM CHAPS, 5 mM DTT) for 30 min on ice at the concentration of 10^7^ cells per 100 µL buffer. Cells were then spun for 30 min at 16,000× *g*, 4 °C. Protein lysate concentrations were measured based on the Bradford assay method using the Cayman Chemical protein determination kit (Cayman Chemical Company, Ann Arbor, MI, USA) based on the manufacturer’s protocol. Assay was performed on a 96-well micro-titer plate by adding equal amount of protein lysate (~100 µg) into wells containing the assay buffer (20 mM HEPES pH 7.4, 0.1% CHAPS, 5 mM DTT, 2 mM EDTA). Reaction was started by the addition of 10 µL of substrate Ac-DEVD-pNA (2 mM). The effect of Ac-DEVD-CHO, a caspase-3 inhibitor was also studied simultaneously on caspase-3 activity. Ten microliter of the inhibitor (200 µM) was added into the wells containing the protein lysate, assay buffer and the substrate. Caspase-3 activity was measured upon the release of pNA molecule, a pale yellow colored product using the micro-plate ELISA reader (Bio-Rad) at the wavelength of 405 nm. Relative caspase-3 activity was expressed as absorbance of enzyme activity compared with the control (untreated cells/DMSO only) based on the equation below:

$$\text{Relative caspase }3\text{ activity}=\;\frac{\text{Absorbance of treated cells }}{\text{Absorbance of control}}$$

### 3.7. Detection of Nucleosomes by Enzyme-Linked Immunosorbent Assay (ELISA)

To confirm the presence of histone-bound DNA fragments (free nucleosomes) in the prostate cancer cells after the treatment of MS17, Cell Death Detection ELISA^PLUS^ kit (Roche Diagnostics) was used according to the manufacturer’s protocol. Cells were plated on a bottom flat tissue culture plates at a concentration of 10^5^ cells per well and incubated for 24 h to allow the cells to adhere. After overnight incubation, MS17 at various concentrations (EC_50_, 2 × EC_50_, 3 × EC_50_) were added to a final volume of 100 µL per well and further incubated for 24, 48 and 72 h. The plate was centrifuged at 600 rpm at 4 °C for 10 min. The cell pellet containing the apoptotic bodies was resuspended in lysis buffer and incubated for 30 min at room temperature. The supernatant and cell lysate solutions (20 µL) were placed in triplicates into wells of streptavidin-coated micro-plate, to which was added 80 µL of the immunoreagent containing a mixture of anti-histone-biotin and anti-DNA-POD. The plate was covered with an adhesive cover foil and incubated for 2 h at room temperature in a shaking incubator at 300 rpm. The unbound antibodies were washed with incubation buffer. The amount of nucleosomes retained by the POD in the immunocomplex, corresponding to the extent of apoptosis and necrosis, was determined with 2,2'-azinobis-3-ethyl-benzothiazoline-6-sulfonic acid (ABTS) as substrate using a micro-plate ELISA reader at a wavelength of 405/490 nm. The amount of enrichment of mono- and oligonucleosomes released into the cytoplasm was calculated using the following formula:

$$\text{Enrichment factor}=\;\frac{\text{Absorbance of sample}}{\text{Absorbance of control}}$$

### 3.8. Statistical Analysis

Results were presented as mean ± standard deviation (SD). All samples were measured in triplicates from each independent experiment. Comparison between sets of data was performed using one-way analysis of variance (ANOVA) followed by Dunnett’s multiple group comparison test. Statistical significant differences between groups was accepted at *p* < 0.05. All statistical analyses were performed using GraphPad Prism version 5.04 (GraphPad Software Inc., La Jolla, CA, USA).

## 4. Conclusions

In conclusion, 1,5-bis(2-hydroxyphenyl)-1,4-pentadiene-3-one (MS17) demonstrated greater inhibitory effects on prostate cancer cell growth and was capable of triggering cell death through apoptosis induction in a dose and time-dependent manner. MS17 might mediate apoptosis through the activation of the caspase-3 intrinsic signaling pathway and this correlates with the morphology and DNA fragmentation analysis. Therefore, we believe that MS17 has the potential to be used as an anti-cancer agent for androgen-independent prostate cancer. However, further investigations are warranted to further confirm its therapeutic effecte and to determine the mechanism of action of this potential diarylpentanoid.
